# Spontaneous Diffusion of an Effective Skin Cancer Prevention Program Through Web-Based Access to Program Materials

**Published:** 2010-10-15

**Authors:** Dawn M. Hall, Cam Escoffery, Eric Nehl, Karen Glanz

**Affiliations:** Centers for Disease Control and Prevention, Division of Cancer Prevention and Control; Emory University, Atlanta, Georgia; Emory University, Atlanta, Georgia; University of Pennsylvania, Philadelphia, Pennsylvania. At the time of this study, Dr Glanz and Ms Hall were affiliated with Emory University

## Abstract

**Introduction:**

Little information exists about the diffusion of evidence-based interventions, a process that can occur naturally in organized networks with established communication channels. This article describes the diffusion of an effective skin cancer prevention program called Pool Cool through available Web-based program materials.

**Methods:**

We used self-administered surveys to collect information from program users about access to and use of Web-based program materials. We analyzed the content of e-mails sent to the official Pool Cool Web site to obtain qualitative information about spontaneous diffusion.

**Results:**

Program users were dispersed throughout the United States, most often learning about the program through a Web site (32%), publication (26%), or colleague (19%). Most respondents (86%) reported that their pool provided educational activities at swimming lessons. The *Leader's Guide* (59%) and lesson cards (50%) were the most commonly downloaded materials, and most respondents reported using these core items sometimes, often, or always. Aluminum sun-safety signs were the least frequently used materials. A limited budget was the most commonly noted obstacle to sun-safety efforts at the pool (85%). Factors supporting sun safety at the pool centered around risk management (85%) and health of the pool staff (78%).

**Conclusion:**

Diffusion promotes the use of evidence-based health programs and can occur with and without systematic efforts. Strategies such as providing well-packaged, user-friendly program materials at low or no cost and strategic advertisement of the availability of program materials may increase program use and exposure. Furthermore, highlighting the benefits of the program can motivate potential program users.

## Introduction

Skin cancer is a serious, growing, and preventable condition (1,2). The *Guide to Community Preventive Services* recommends interventions in outdoor recreation settings as effective strategies to reduce exposure to ultraviolet radiation and help prevent skin cancer (3,4). The Web site for the National Cancer Institute's Research-tested Intervention Programs describes at least 10 proven interventions to promote sun safety and prevent skin cancer (rtips.cancer.gov/rtips/index.do). Diffusion of such interventions allows them to broadly affect public health.

According to the Diffusion of Innovations model, diffusion is the process by which an innovation is communicated through certain channels over time among members of a social system (5,6). Dissemination refers to planned, systematic efforts designed to make a program more widely available to a target audience (6). Active dissemination methods may include packaging a program, establishing linkage systems, building organizational capacity, and providing technical assistance for implementation (7-9). The establishment of linkage systems and linking agents connects program users to program developers and can enhance the implementation and sustainability of a program (10). However, spontaneous diffusion can also occur in the absence of active dissemination efforts (11,12). Characteristics of an innovation, including its relative advantage, compatibility, complexity, and the ease with which it can be tried and observed, affect rates of both spontaneous diffusion and active dissemination efforts (6). Little information exists about spontaneous diffusion of effective interventions.

Pool Cool is an environmental and educational sun-safety program designed for use at outdoor swimming pools. The target audience is children aged 5 to 10 years who are enrolled in swimming lessons. A pilot was tested in 1998 and has been evaluated in a randomized, controlled trial (1999), a dissemination pilot study (2000-2002), and a diffusion trial (2003-2006). Pool Cool has had substantial positive effects on child sun protection behaviors and sunburn rates, parent sun protection behaviors, lifeguard sunburn rates, and the sun protection programs, policies, and environments at outdoor pools (13-16). In addition to program implementation occurring at study pools, program awareness and use has spread to nonstudy pools through various communication channels.

This article describes the extent and nature of the spontaneous diffusion of the Pool Cool program during a 4-year period. We describe the processes through which spontaneous diffusion has occurred, access to and use of Web-based program materials, levels of program adoption and implementation among Web registrants, and supporting factors and obstacles to program implementation.

## Methods

We conducted the Pool Cool Diffusion Trial (15) nationally from June through August for 4 consecutive summers (2003-2006). Recruitment procedures are detailed elsewhere (15). Recruited pools were both public and private and were required to be outdoor, offer swim lessons to children aged 5 to 10 years, and have enough parent/child patrons to recruit at least 20 parents to complete surveys. During the study, we provided participating pools with educational materials, including 8 sun-safety lessons, an illustrated flip book to make the lessons more engaging and interactive, and a *Leader's Guide* to the Pool Cool program. We also provided poolside activities to complement the lessons, including ultraviolet index activity cards, a Play it Safe in the Sun poster, and a sun-safety-themed *Jeopardy!*-style game board. We also gave pools a *Decision Maker's Guide for Sun Safety*, a gallon jug of sunscreen, aluminum sun-safety signs, and small incentive items.

We made these materials available year-round to nonstudy pools through the Pool Cool and National Recreation and Park Association (NRPA) Web sites. On the Pool Cool Web site, materials could be downloaded as portable document files (PDFs) and reproduced for use at the pool. A Pool Cool e-mail address was provided on the Web site so that incentive items could be requested and purchased. Additional materials identical to those provided to study pools also could be purchased through the NRPA Web site.

Before downloading or purchasing materials from the Web sites, people from nonstudy pools were required to register online, provide contact information, and agree to be contacted about how their pool used the program materials. At the end of each summer, we sent registrants a survey about Pool Cool Web-based materials. We used data from the Web-based surveys and surveys from the diffusion study to compare study pools with nonstudy pools that accessed the materials online. We collected additional information about the spontaneous diffusion through e-mails sent to the official Pool Cool e-mail address during the diffusion study. We included only e-mails from people who inquired about the Pool Cool program and who had not participated in a Pool Cool research study. All data collection procedures were approved by the University of Hawaii Committee on Human Studies (CHS no. 11575) and the institutional review board at Emory University (IRB no. 156-2004).

The registration form for obtaining Web-based materials asked registrants for their name, organization, contact information, type of organization at which they planned to use the Pool Cool materials, the ages and number of children expected to be exposed to the program, and how they learned about the program. The survey about Web-based materials sent to registrants at the end of the summer included questions about pool characteristics, use of materials, environmental and organizational supports for sun safety at the pool, and obstacles and supporting factors for sun-safety efforts at the pool (Appendix).

Questions about pool characteristics included community description (urban, suburban, or rural), weekly pool attendance, and number of staff. We included these questions on baseline pool manager surveys as well, allowing for comparison of the diffusion study pools with nonstudy pools that registered for program materials online.

We used SPSS version 16.0 (SPSS, Inc, Chicago, Illinois) to conduct all quantitative analyses. We used χ^2^ tests to compare people who completed the survey about Web-based materials with people who completed only the registration form and to compare diffusion study pools with nonstudy pools. We computed frequencies to assess use of program materials, levels of program implementation, and the importance of supporting factors and obstacles to sun safety at the pool. Some respondents returned more than 1 follow-up survey, so we conducted these analyses twice: once using the responses from each respondent's first survey and a second time using each respondent's highest response to each survey item across all returned surveys. Conducting analyses using each respondent's highest response allowed us to determine whether respondents *ever* used each program component and provided a summary of the highest levels of program use that occurred at all pools.

We used qualitative methods to analyze e-mail messages related to spontaneous diffusion that were sent to the official Pool Cool e-mail address. We received e-mails related to spontaneous diffusion from 11 people. One researcher reviewed the e-mails for thematic topics and categorized the e-mails into themes. A second researcher then independently categorized the e-mails into the thematic categories, and the study team discussed any discrepancies until consensus was achieved. The thematic categories were not mutually exclusive, and some e-mails were captured by more than 1 theme.

## Results

Of the 291 Web registrants (representing 291 different pools), most were aquatics or recreation managers, supervisors, coordinators, or directors (88%). A small number were health professionals, researchers or educators (8%), and lifeguards or swimming instructors (5%). Most registrants reported that the materials would be used at a public pool (80%) and expected at least 100 children to be exposed to program materials (70%). Pools were dispersed nationally in states with (n = 28) and without (n = 12) study pools, and 3 pools were in 2 Canadian provinces. All registrants reported that children between the ages of 5 and 10 years would be exposed to the program, and nearly 80% reported that younger and older children also would be exposed. Registrants reported hearing about the program through a Web site (32%), publication (26%), friend or colleague (19%), through NRPA (12%), or at a conference (11%).

Ninety-five Web registrants (33%) returned at least 1 survey about Web-based materials. Twenty-five completed a survey 2 years in a row, and 7 completed a survey 3 years in a row. Survey completers were more likely to report plans to use the materials at a public pool than in other settings (eg, private swimming pools) (89.2% vs 74.9%, χ^2^ = 8.29, *P* = .005) and were more likely to anticipate that 100 or more children would be exposed to the materials (79.6% vs 67.1%, χ^2^ = 5.03, *P* = .03). No other significant differences were found between Web registrants who completed at least 1 survey and Web registrants who did not complete a survey.

Web registrants were equally distributed between northern and southern latitudes and tended to be in rural or suburban areas (68%). Most respondents reported that fewer than 2,000 patrons came to the pool each week, but the pools had large staff sizes; more than 60% reported a staff of 23 or more. The only significant difference between diffusion study pools (n = 466) and nonstudy pools that accessed materials online was related to staff size, with nonstudy pools more likely to have a staff of 23 or more (Table 1).

### Access to program materials and levels of implementation

Some respondents used additional program materials after the first summer they accessed the materials (Table 2). The *Leader's Guide* and lesson cards, which are core program materials, were the most frequently used items. Many respondents reported using multiple items. More than 37% used 3 to 7 Pool Cool items, and nearly 30% accessed 8 or more of the items available online. Only 18% of respondents reported that they did not access any of the items. A higher percentage of respondents who accessed the sunscreen tips poster and the aluminum sun-safety signs reported using each item often, usually, or always compared with other items (Table 3).

A minority of respondents reported having additional supports for the program. One-third of respondents reported that their pool added shade structures or shaded areas to the pool grounds during the summer. Just over 15% reported that their pool developed or purchased additional resources to complement Pool Cool materials, and only 3% reported that their pool received outside sponsorship or recognition for the Pool Cool program. Most respondents (86%) reported that their pool provided educational activities in swimming lessons. Most respondents indicated that their pools provided programs and policies for sun safety among the pool staff (92%) and swimmers and patrons (73%).

### Supporting factors and obstacles

When asked about supporting factors for sun safety at the pool, most respondents rated health concern (79%) and risk management of employees (ie, identifying, addressing, and minimizing risks associated with working at the pool to avoid additional costs to the pool, 85%) as very important, and community relations (88%) and community or citizen demand (73%) as at least somewhat important. Overall, respondents rated obstacles to sun safety as slightly less important than supporting factors. Most respondents rated as somewhat or very important limited money, budget, or staff (85%); the design of the pool facility (63%); and lack of information or guidance (59%). Only 47% of respondents reported that sun safety not being a priority or a concern was a somewhat or very important obstacle.

### E-mail themes

Themes of e-mails sent to the Pool Cool e-mail address related to inquiries about program materials, general program information, program evaluation, and speaking engagement and were divided into 2 categories. Most people who contacted the Pool Cool Web site (70%) were interested in obtaining program materials for use in program implementation, a sun-safety event, or for general sun-safety information. A few requested general information about the program; 1 person was interested in the evaluation survey, 1 person wanted to use the program as an example in a graduate class on marketing, and 1 person wanted a speaker to make a presentation about the program.

## Discussion

This article describes the extent and nature of program diffusion that occurred at pools in comparison with those participating in the Pool Cool Diffusion study. Most Web registrants were referred to the Pool Cool intervention materials from a Web site, publication, or colleague, and the use of the Pool Cool materials spread to many states other than the states of Pool Cool diffusion study pools. Our results indicate that diffusion of Web-based program materials occurred outside of the active dissemination efforts that were used and evaluated in the Pool Cool Diffusion Trial. Informal linkages and communication channels may have facilitated program diffusion to nonstudy pools.

Respondents downloaded various program materials, and use of downloaded materials was high, especially the use of the *Leader's Guide* and lesson cards. These materials are core components of the program and are critical to implementation. An "intervention package" allowed community organizations to implement research-tested interventions (9,17). All program components were packaged together in a kit, and materials were presented in nonacademic language in a user-friendly manual (18). Although the Web-based materials could be accessed individually, all the materials were available in 1 location online with user-friendly instructions for program implementation at their pool (*Leader's Guide*). The availability of the Pool Cool materials and instructions on the Web site likely facilitated program implementation and use at nonstudy pools. Although respondents may not have used all elements of the program, exposure to the program seems to have increased their usability and use.

Fewer respondents reported reproducing or purchasing program materials than they did downloading materials. Pools may not have the funds to reproduce or purchase program materials but can still benefit from access to downloadable PDFs online. For example, the *Leader's Guide* can be reviewed and referenced on a computer and provide information without being printed.

The most common supporting factors for sun-safety efforts at the pools were risk management and health of employees and community relations. Highlighting the benefits and the advantage of implementing a new program may be a way to motivate potential program users (5,6). Furthermore, the intended outcomes of the program should be compatible with the values and needs of the intended audience (5,6). The most common obstacle to sun-safety efforts at the pools was limited resources. Making program materials available online at a low or no cost allowed users to try out the program before making a commitment to adopt the program. A recent study of another Web-based sun-safety program also found that Internet-based, interactive, educational programs can increase public participation in existing health-promotion campaigns (19).

We reported data on spontaneous diffusion e-mails to supplement the information collected from the Web-based materials surveys. Data from both sources suggested that access to program materials does not always result in program implementation. Sometimes program materials were used solely for a specific pool event or adapted to fit the specific needs of the pool, and pools were not typically implementing all components of the program. This fact illustrates that program diffusion does not always occur with high fidelity, and practical use of an innovation may differ from the original intent of the developer. However, if the full program is not adopted, exposure to the health promotional materials may still provide some benefits.

This study has several limitations. The information was based on self-report and may be subject to biases, such as inaccurate recall, misrepresentation, and social desirability. Second, many registered users did not complete the survey, which limits the generalizability of the study results. However, our low response rates are similar to those of other Web-based strategies for the diffusion of sun-safety interventions (20). On the basis of anecdotal evidence, we believe that the survey responses underestimate the use of Pool Cool materials. People who obtained the Web-based materials were not participating in the diffusion study and likely were more focused on practice-based implementation rather than evaluation. This is a conundrum of trying to collect data from people who chose not to participate in a scientific study. Those people who did complete a survey typically planned to use the program at a public pool where many children would be reached through the program. The program is ideal for this type of pool setting because of the potential for large reach and may be more compatible with the needs of larger outdoor pools. Smaller, privately owned pools may have implemented the program in a different way than did large, public pools. Furthermore, the program may not be compatible with a smaller pool setting. However, drawing clear conclusions about such differences in program use is beyond the scope of this study. Another limitation is the online registration process. The program may have been used at pools that were not registered on the Web site. However, we do not have information about these pools and cannot determine how they differ from pools that registered and accessed the materials online.

The Pool Cool program was designed to be easy to use and compatible with the sun-safety needs of large pools, and the program can easily be tried before an organization commits to adopt it. Furthermore, we created user-friendly materials and made them available to the public online at no cost. These characteristics were key to program diffusion, and the study results demonstrate that diffusion of the Pool Cool program occurred outside of active dissemination efforts (5,6).

Both active dissemination and spontaneous diffusion can promote the use of science-based programs in communities (21-23). Future research should further explore factors leading to this type of diffusion and how organizations adopt and implement program components. When developing new programs, researchers should ensure that the attributes of the program are designed to maximize diffusion rates.

## Figures and Tables

**Table 1:** Characteristics of Web Registrants Compared With Pools Participating in the Pool Cool Diffusion Trial, 2003-2006

**Characteristic**	Overall (N = 561), n (%)^a^	Web Registrants (N = 95), n (%)^a^	Study Pools (N = 466), n (%)^a^	χ^2^, *df*	*P* Value
**Latitude**					
North	274 (48.8)	46 (48.4)	228 (48.9)	.008, 1	.93
South	287 (51.2)	49 (51.6)	238 (51.1)		
**Community description**					
Urban	204 (36.4)	26 (27.4)	178 (38.2)	3.14, 1	.08
Rural/suburban	351 (62.6)	65 (68.4)	286 (61.4)		
**Weekly pool attendance**					
<1,000	228 (40.6)	38 (40.0)	190 (40.8)	.007, 2	.99
1,000-1,999	172 (30.7)	29 (30.5)	143 (30.7)		
≥2,000	159 (28.3)	27 (28.4)	132 (28.3)		
**Total number of pool staff**					
1-10	176 (31.4)	12 (12.6)	164 (35.2)	28.7, 2	<.001
11-22	161 (28.6)	23 (24.2)	138 (29.6)		
≥23	221 (39.4)	59 (62.1)	162 (34.8)		

a Percentages may not total 100 because of missing responses.

**Table 2:** Percentage of Web Survey Respondents (N = 95) Who Downloaded, Purchased, or Reproduced Materials,^a^ Pool Cool Diffusion Trial, 2003-2006

Materials	First Survey		Ever (Across All Surveys^b^)	

	Downloaded, N (%)	Purchased or Reproduced, N (%)	Downloaded, N (%)	Purchased or Reproduced, N (%)
Pool Cool *Leader's Guide*	56 (59)	24 (25)	62 (65)	28 (30)
Pool Cool lesson cards	48 (50)	21 (22)	55 (58)	24 (25)
Play it Safe in the Sun poster	46 (48)	16 (17)	53 (56)	20 (21)
Sunscreen tips poster	40 (42)	21 (22)	51 (54)	26 (27)
*Mini Big Book* illustrations	42 (44)	15 (16)	47 (50)	20 (21)
UV Index activity cards	37 (39)	16 (17)	45 (47)	21 (22)
*Decision -Maker's Guide for Sun Safety*	33 (35)	15 (16)	40 (42)	17 (18)
*Jeopardy!*-style game board	30 (32)	10 (10)	35 (37)	10 (10)
Aluminum sun-safety signs	25 (26)	8 (8)	28 (30)	11 (12)

a The options of downloading, reproducing, and purchasing program materials are not mutually exclusive.

b Some respondents completed a follow-up survey in more than 1 year from 2003 to 2006.

**Table 3:** Frequency of Use of Program Materials, Pool Cool Diffusion Trial, 2003-2006^a^

**How often did your pool . . .**	Usually/Always or Often, n (%)
Display the sunscreen tips poster? (N = 56)	32 (57)
Display aluminum sun-safety signs? (N = 33)	15 (46)
Use the *Mini Big Book* illustrations? (N = 49)	17 (35)
Conduct any of the poolside activities? (N = 64)	22 (34)
Use the Pool Cool *Leader's Guide*? (N = 64)	17 (27)

a Only respondents who reported downloading, purchasing, or reproducing the item are included.

## Appendix

### Items and Response Options on Survey About Pool Cool Web-Based Materials

**Table:**

**Survey Item**	Response Options
**Did you or someone at your pool download and/or produce any of the following items?**	
1. Pool Cool *Leader's Guide*	Downloaded (Y/N) Produced (Y/N)
2. Pool Cool lesson cards	
3. *Mini Big Book* illustrations	
4. UV Index activity cards	
5. Play it Safe in the Sun poster	
6. *Jeopardy!*-style game board	
7. Aluminum sun-safety signs	
8. Sunscreen tips poster	
9. *Decision-Maker's Guide for Sun Safety*	
**This summer, how often did your pool . . .**	
10. Use the Pool Cool *Leader's Guide*	1 = Rarely/Never 2 = Sometimes 3 = Often 4 = Usually/Always
11. Use the *Mini Big Book* illustrations to help teach sun protection lessons?	
12. Conduct any of the poolside activities?	
13. Display the sunscreen tips poster?	
14. Display the other aluminum Pool Cool sun-safety signs in the pool area?	
**15. Did your pool add any shade structures or shaded areas to the pool grounds this summer?**	Y/N (If yes, please describe)
**16. Did your pool develop or purchase additional resources to complement the Pool Cool materials you downloaded or modify any of the Pool Cool materials for use at your pool?**	
**17. Did your pool receive outside sponsorship in the form of money or materials to enhance or add to the Pool Cool program this summer?**	
**18. Did your pool receive any recognition, including awards and/or media coverage, for the Pool Cool skin cancer prevention program this summer?**	
**19. What best describes the community where the pool is located?**	Urban, suburban, or rural
**20. Approximately how many people are admitted to the pool each week during the summer?**	1 = <500 2 = 500-999 3 = 1,000-1,499 4 = 1,500-1,999 5 = ≥2,000
**21. How many aquatic staff work at your pool during the summer?**	Full time: Part time:
**22. How many staff at this pool are seasonal (summer only)?**	1 = None 2 = Few/some 3 = Most 4 = All
**23. How many years have you been working at this pool?**	(respondent filled in)
**24. How often does your pool support and/or sponsor special events/activities such as water safety days, public holiday celebrations, etc?**	1 = Never 2 = Occasionally 3 = Sometimes 4 = Often
**25. How much does your recreation department (supervisory organization) support your efforts/activities?**	1 = Not at all 2 = A little/somewhat 3 = Mostly 4 = Completely
**In your aquatics program, indicate whether you provide each of these types of programs about sun safety and/or skin cancer prevention:**	
26. Programs/policies for lifeguards/staff?	1 = Rarely or never 2 = Sometimes 3 = Often 4 = Usually/always
27. Programs/policies for swimmers/patrons?	
28. Educational activities in swimming lessons?	
29. Has Pool Cool been conducted at your pool before?	1 = Not sure 2 = No 3 = Yes
**Indicate how often your pool implements each of these policies, environments, and/or programs for its patrons/visitors/users:**	
30. Schedule classes/events to avoid peak sun hours?	1 = Never, not planning 2 = Never, but planning 3 = Sometimes 4 = Often 5 = Usually/always
31. Sell or provide sunscreen?	
32. Sell or provide other protective items?	
33. Post signs about sun safety?	
34. Post daily UV ratings?	
35. Provide sun-safety educational materials?	
**How important is each of these supporting factors for your choices or plans regarding sun safety at your pool?**	
36. Health concern	1 = Not at all important 2 = Not very important 3 = Somewhat important 4 = Very important
37. Risk management of employees	
38. Community/citizen demand	
39. Community relations	
**How important is each of these potential obstacles for your choices or plans regarding sun safety at your pool?**	
40. Limited money/budget/staff	1 = Not at all important 2 = Not very important 3 = Somewhat important 4 = Very important
41. Lack of information and guidance	
42. How the pool facility is designed	
43. Not a priority concern	
